# Melatonin protects against oxybenzone-induced deterioration of mouse oocytes during maturation

**DOI:** 10.18632/aging.202323

**Published:** 2020-12-29

**Authors:** Long Jin, Hai-Ying Zhu, Xiang-Jin Kang, Li-Ping Lin, Pu-Yao Zhang, Tao Tan, Yang Yu, Yong Fan

**Affiliations:** 1 Department of Gynecology and Obstetrics, Key Laboratory for Major Obstetric Diseases of Guangdong Province, Key Laboratory of Reproduction and Genetics of Guangdong Higher Education Institutes, The Third Affiliated Hospital of Guangzhou Medical University, Guangzhou 510150, Guangdong, China; 2 Beijing Key Laboratory of Reproductive Endocrinology and Assisted Reproductive Technology and Key Laboratory of Assisted Reproduction, Ministry of Education, Center for Reproductive Medicine, Department of Obstetrics and Gynecology, Peking University Third Hospital, Beijing 100191, China; 3 Yunnan Key Laboratory of Primate Biomedical Research, Institute of Primate Translational Medicine, Kunming University of Scienceand Technology, Kunming 650500, Yunnan, China

**Keywords:** melatonin, oxybenzone, oocyte maturation, oocyte quality, mouse

## Abstract

Oxybenzone (OBZ), an ultraviolet light filter that is widely used in sunscreens and cosmetics, is an emerging contaminant found in humans and the environment. Recent studies have shown that OBZ has been detected in women’s plasma, urine, and breast milk. However, the effects of OBZ exposure on oocyte meiosis have not been addressed. In this study, we investigated the detrimental effects of OBZ on oocyte maturation and the protective roles of melatonin (MT) in OBZ-exposed mouse models. Our *in vitro* and *in vivo* results showed that OBZ suppressed oocyte maturation, while MT attenuated the meiotic defects induced by OBZ. In addition, OBZ facilitated H3K4 demethylation by increasing the expression of the Kdm5 family of genes, elevating ROS levels, decreasing GSH, impairing mitochondrial quality, and disrupting spindle configuration in oocytes. However, MT treatment resulted in significant protection against OBZ-induced damage during oocyte maturation and improved oocyte quality. The mechanisms underlying the beneficial roles of MT involved reduction of oxidative stress, inhibition of apoptosis, restoration of abnormal spindle assembly and up-regulation of H3K4me3. Collectively, our results suggest that MT protects against defects induced by OBZ during mouse oocyte maturation *in vitro* and *in vivo*.

## INTRODUCTION

The incidence of skin cancer has increased worldwide over the past decades, which has resulted in substantial health and economic burdens [1, 2]. With increasing awareness regarding skin cancer, more than approximately 10,000 tons of ultraviolet (UV) filters are produced for the global market annually and are used to protect skin from direct sunlight [3]. Oxybenzone (2-hydroxy-4-methoxyphenyl-phenylmethanone, OBZ), also known as benzophenone-3, is an organic UV filter present in plastic surface coatings, sunscreen and personal care products, including lip balm, lotion, hair sprays, and shampoo [4]. Due to the increase in use of OBZ, it has become a ubiquitous environmental contaminant. Some studies have reported that OBZ has been detected in seawater, rivers, lakes, wastewater, swimming pools, and even drinking water [5–7]. Furthermore, because OBZ can be readily absorbed by human skin, detectable levels of OBZ have been found in human urine, breast milk, serum, cord and blood [8, 9]. Recently, Matta et al. (2019) showed that the geometric mean maximum plasma concentration of OBZ ranged from 740 nM (169.3 ng/mL) to 920 nM (209.6 ng/mL), which exceeded the FDA level for toxicological concern (0.5 ng/mL or 2.2 nM) [10]. Therefore, it is necessary to further investigate the potential health risks of OBZ exposure.

Numerous studies have shown the negative effects of OBZ on the reproductive system, where it acts as an endocrine disrupting chemical (EDC) [11–13]. In fish, OBZ exposure caused a reduction in egg production and induced egg protein production in males and juveniles [14, 15]. One prior study by Balazs et al. (2016) showed that OBZ exposure increased mortality and developmental disorders in zebrafish embryos [16]. In rodents, OBZ reduced body weight and uterine weight, increased abnormal sperm levels, decreased epididymal sperm density, altered gland morphology and function, prolonged the estrous cycle, and induced idiopathic sudden death in lactating mothers [6, 11, 17, 18]. A recent *in vitro* study showed that exposure to OBZ (5.8 nM) decreased the total number of oocytes, the number of nests per ovary and the early primary follicle population, indicating that OBZ perturbed the early events of germ cell development in rat whole ovary cultures [19]. In humans, OBZ exposure was associated with pregnancy duration, body weight, head circumference, fetal growth, and Hirschsprung’s disease (HSCR) [13, 20, 21]. Because of the potential risks of OBZ, it has been banned in Hawaii, Florida, US Virgin Islands, Palau, Bonaire, and nature reserve sites in Mexico [22, 23]. To our knowledge, there have been no reports on the toxic effects of OBZ on mouse oocyte maturation, and its specific mechanisms remain unknown.

Melatonin (5-methoxy-N-acetyltryptamine, MT) is a circadian hormone released by the pineal gland that carries out diverse functions due to its antioxidative [24], antiapoptotic [25], and many other critical properties [26]. MT has been found in human preovulatory follicular fluid [27, 28], where free oxygen radicals are in high concentrations during follicular growth [29]. Furthermore, trials involving the clinical application of MT for infertile women have reported improved outcomes resulting from the use of assisted reproductive technology (ART) [30]. The exact molecular mechanism of MT in the ovary, follicular fluid, and oocytes has not been fully elucidated, but numerous studies have shown that MT protects against defects induced by EDC during oocyte maturation and improves oocyte and embryo quality [24, 31].

The objective of this study was to determine whether OBZ exposure affected critical events during mouse meiotic maturation, including the first polar body extrusion (PBE), histone modifications, oxidative stress, early apoptosis, mitochondrial quality, and spindle assembly. We first investigated whether MT supplementation could protect against OBZ-induced damage during oocyte maturation. Our results showed that OBZ exposure altered histone modifications and caused oxidative stress, which was followed by early apoptosis, decreased mitochondrial quality, and disrupted spindle organization. MT effectively restored the abnormal levels of trimethyl-histone H3 lysine 4 (H3K4me3), suppressed oxidative stress, reduced the amount of early apoptosis, and ameliorated the deterioration induced by OBZ exposure in mouse oocytes.

## RESULTS

### Effects of melatonin on *in vitro* development of OBZ-exposed mouse oocytes and embryos

**Figure 1:**
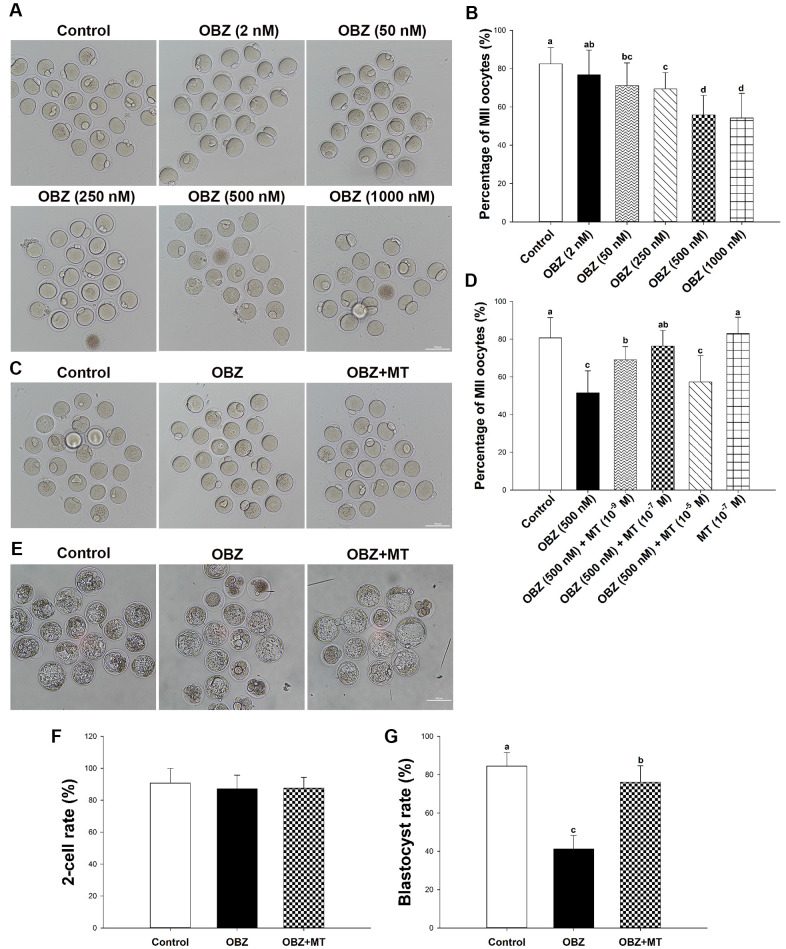
**Effects of melatonin on mouse oocytes and embryos following OBZ exposure *in vitro*.** (**A**) Representative images of oocytes that extruded the first polar body (PB1) in the control and OBZ-exposed groups. Scale bar, 100 μm. (**B**) Polar body extrusion (PBE) rate in different treatment groups. (**C**) The mouse oocyte morphologies in the control, OBZ, and OBZ+MT groups. Scale bar, 100 μm. (**D**) The effects of gradient concentrations of melatonin on the rate of PBE in OBZ-exposed oocytes. (**E**-**G**) Mouse embryo morphologies (**E**) and embryo development rate (**F**, **G**) from the 2-cell to blastocyst stages in the control, OBZ, and OBZ+MT groups. Values indicated by different letters are significantly different (P < 0.05). Control, untreated control group; OBZ, oxybenzone-exposed group; OBZ+MT, “oxybenzone + melatonin” treatment group.

To investigate the toxic effects of OBZ exposure on the *in vitro* maturation (IVM) of immature oocytes retrieved from ICR mice, we first cultured oocytes with increasing concentrations (2, 50, 250, 500, and 1000 nmol/L) of OBZ. As shown in Figure 1A, 1B, in the control group, most of the oocytes released the first polar body (82.45 ± 8.54%, n = 367). However, OBZ exposure significantly decreased the PBE rate of mouse oocytes (25 nM, 74.99 ± 9.38%, n = 391, P < 0.05; 50 nM, 71.11 ± 11.87%, n = 346, P < 0.05; 100 nM, 71.19 ± 4.89%, n = 334, P < 0.05; 250 nM, 69.41 ± 8.59%, n = 314, P < 0.05; 500 nM, 55.87 ± 10.19%, n = 351, P < 0.05; and 1000 nM, 54.21 ± 12.68%, n = 343, P < 0.05; Figure 1B). When the OBZ concentration was 2 nM, there was no significant difference in the maturation rate between the 2 nM OBZ-exposed oocytes and untreated oocytes (76.84 ± 12.84%, n = 360 vs 82.45 ± 8.54%, n = 367, P = 0.10). These results suggested that OBZ exposure inhibited the meiotic maturation of mouse oocytes in a dose-dependent manner. Based on the previous study [10], 500 nmol/L OBZ was used in further treatment experiments.

Next, to investigate whether MT has protective effects against OBZ-induced meiotic arrest, MT was dissolved in absolute ethanol and then diluted in 500 nmol/L OBZ IVM medium to yield a final concentration of 1 × 10^−9^ mol/L, 1 × 10^−7^ mol/L, and 1 × 10^−5^ mol/L, respectively. The quantitative results showed that 1 × 10^−7^ mol/L MT not only significantly increased the proportion of PBE in OBZ-exposed oocytes (76.33 ± 8.34%, n = 241, P < 0.05; Figure 1C, 1D) compared to that of the treatment with OBZ alone (51.62 ± 11.59%, n = 245), but the 1 × 10^−7^ mol/L MT treatment results were similar to those of the treatment with MT alone (83.03 ± 8.64%, n = 245) and untreated control group (80.72 ± 10.76%, n = 262). Higher concentrations of MT did not obviously improve oocyte maturation in the OBZ exposure group (57.40 ± 13.95%, n = 254). Although 1 × 10^−9^ mol/L MT significantly increased the rate of PBE (69.09 ± 7.01%, n = 220) compared to that of the group treated with only OBZ, it was significantly lower than that of the control group. Based on these results, we chose to use 1 × 10^−7^ mol/L MT for treatment in the subsequent study.

We then asked whether OBZ exposure affected early embryonic development in mice and evaluated the effects of MT on the *in vitro* development of OBZ-treated embryos. As shown in Figure 1E–1G, the proportion of embryos that developed to the blastocyst stage was significantly lower in the group treated with 500 nM OBZ than it was in the control untreated group (41.25 ± 7.04%, n = 191 vs 84.52 ± 7.08%, n = 184, P < 0.05). As expected, 1 × 10^−7^ mol/L MT supplementation effectively improved the developmental competence of OBZ-exposed embryos (87.54 ± 6.78%, n = 217). However, there were no significant differences in the cleavage rates of embryos (87.20 – 90.71%) among the different groups.

### Effects of melatonin on histone methylation levels in mouse oocytes exposed to OBZ

**Figure 2:**
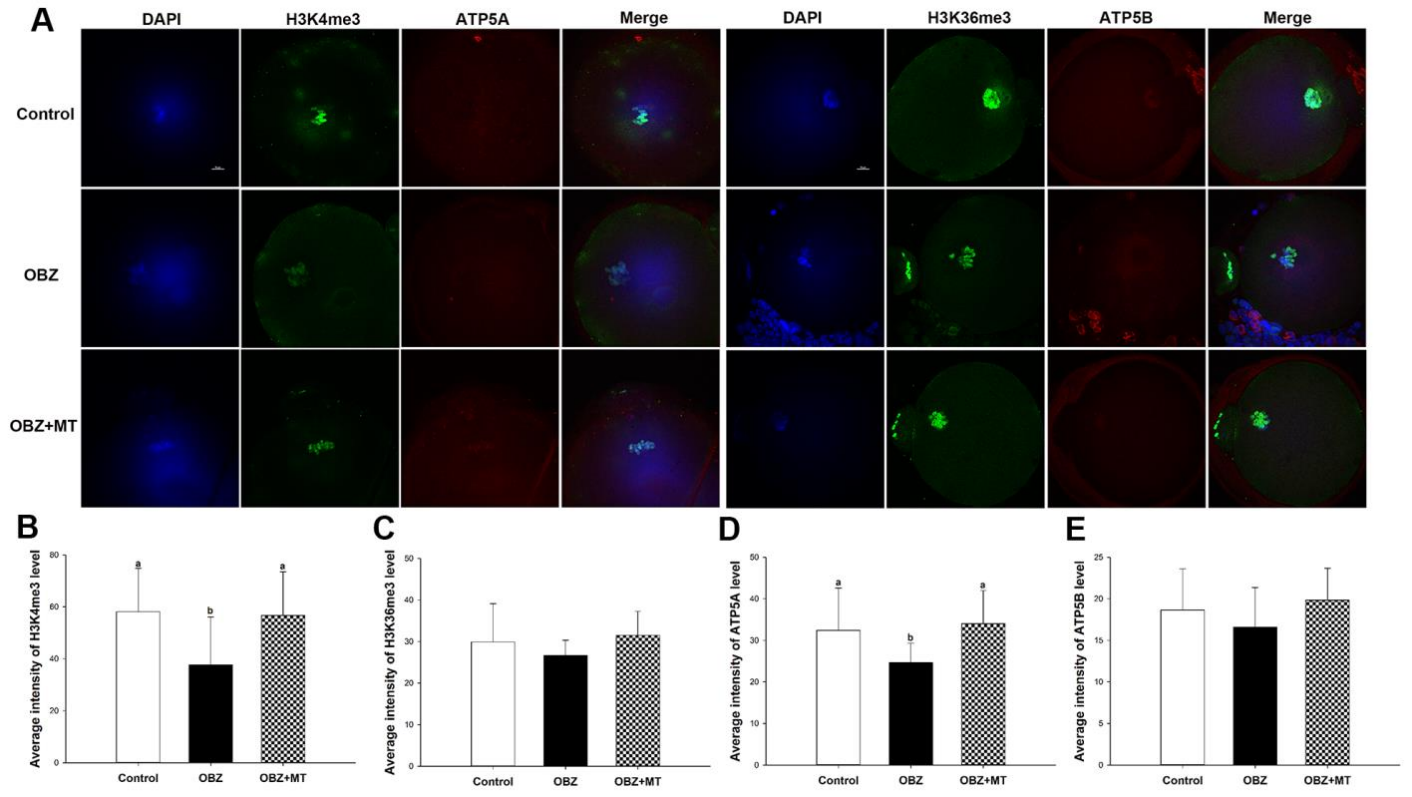
**Effects of melatonin on H3K4me3, H3K36me3, ATP5A, and ATP5B levels in OBZ-exposed oocytes.** (**A**) Immunofluorescence staining for H3K4me3, H3K36me3, ATP5A, and ATP5B in the control, OBZ-exposed, and melatonin+OBZ-treated oocytes. Green, H3K4me3 and H3K36me3; red, ATP5A and ATP5B; blue, DNA. Scale bar, 10 μm. (**B**–**E**) Average fluorescent intensities for H3K4me3 (**B**), H3K36me3 (**C**), ATP5A (**D**), and ATP5B (**E**) in mouse oocytes from the different groups. Values indicated by different letters are significantly different (P < 0.05). The experiments were repeated five times, with n = 15-20 per group. Control, untreated control group; OBZ, oxybenzone-exposed group; OBZ+MT, “oxybenzone + melatonin” treatment group.

Histone modifications play functional roles in oocyte meiotic maturation, fertilization, and further embryonic development in mammals. To determine whether melatonin reversed the OBZ-induced decrease in mouse oocyte maturation by altering histone modifications, the global H3K4me3 and trimethylation of lysine 36 on histone H3 (H3K36me3) levels were examined by immunofluorescence staining. OBZ-exposed oocytes presented significantly lower H3K4me3 fluorescence intensity levels than did those in the control group (P < 0.05; Figure 2A, 2B), while no statistically significant differences were found in the average fluorescence intensity of H3K36me3 among the control, OBZ, and OBZ+MT groups (Figure 2A, 2C). As expected, the detrimental effects of OBZ could be rescued by MT; coincubation with MT effectively increased H3K4me3 fluorescence intensity (P < 0.05). These results suggested that OBZ exposure demethylated trimethyl-histone H3K4 in *in vitro* matured oocytes and that MT could restore these changes.

**Figure 3:**
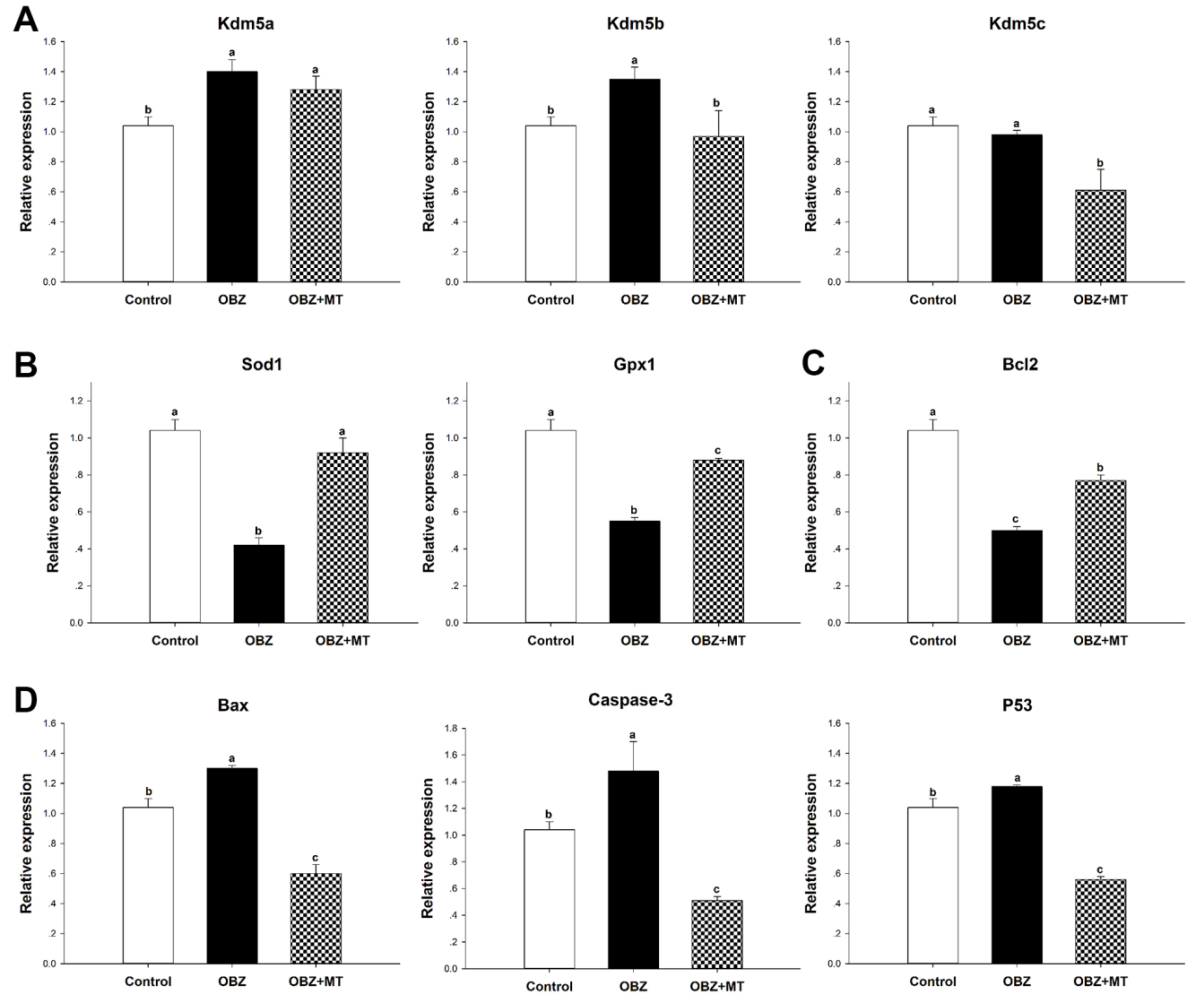
**Effects of 10^-7^ mol/L melatonin with or without 500 nmol/L oxybenzone on gene expression in matured mouse oocytes.** (**A**) Histone H3K4me3 demethylation-related genes (Kdm5a, Kdm5b, and Kdm5c). (**B**) Antioxidative stress genes (SOD and GPx1). (**C**) Anti-apoptosis gene Bcl2. (**D**) Pro-apoptosis genes (Bax, Caspase-3, and P53). Within each category, groups marked with different superscripted letters are significantly different (P < 0.05). The experiments were repeated three times, with n = 80-100 per group. Control, untreated control group; OBZ, oxybenzone-exposed group; OBZ+MT, “oxybenzone + melatonin” treatment group.

To further unravel the possible mechanism underlying the beneficial effects of MT on histone H3K4 trimethylation in OBZ-exposed oocytes, we detected the mRNA levels of Kdm5a, Kdm5b, and Kdm5c, which are H3K4-specific histone demethylases. We found that the transcript levels of Kdm5a and Kdm5b were significantly increased in OBZ-exposed oocytes compared with their controls, but OBZ exposure did not change the expression level of Kdm5c (Figure 3A). In the OBZ+MT group, Kdm5b and Kdm5c mRNA transcript levels were lower than they were in the OBZ group. Somewhat unexpectedly, MT supplementation significantly upregulated Kdm5a gene expression compared with that of the untreated oocytes.

### Effects of melatonin on oxidative stress and early apoptosis in mouse oocytes exposed to OBZ

**Figure 4:**
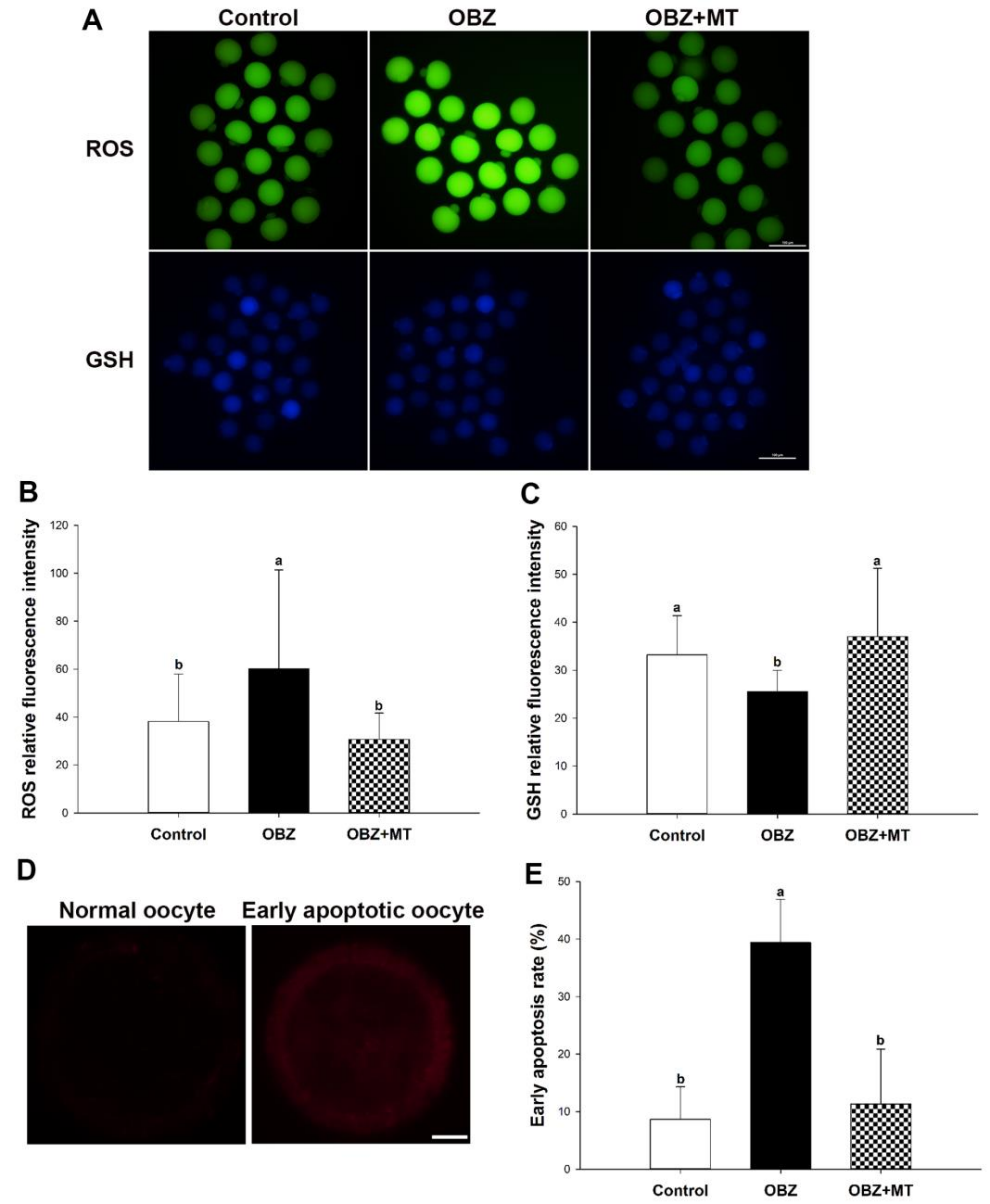
**Effects of melatonin on intracellular reactive oxygen species (ROS) levels, glutathione (GSH) levels, and early apoptosis in oxybenzone-exposed oocytes.** (**A**) Representative images of ROS and GSH levels in the control, OBZ-exposed, and melatonin+OBZ-treated oocytes. Scale bar, 100 μm. (**B**) Intracellular ROS and (**C**) GSH levels in mouse oocytes from each treatment group. (**D**) Representative images of normal oocytes and early apoptotic oocytes. Scale bar, 20 μm. (**E**) Proportion of early apoptotic oocytes in each treatment group. Values indicated by different letters are significantly different (P < 0.05). The experiments were repeated three times, with n = 15-20 per group. Control, untreated control group; OBZ, oxybenzone-exposed group; OBZ+MT, “oxybenzone + melatonin” treatment group.

To investigate whether OBZ exposure induced oxidative stress, we detected the ROS levels in each group. As shown in Figure 4A, 4B, the ROS signal was significantly higher in the OBZ exposure group than they were in the control group (P < 0.05), whereas there was little ROS signal in the OBZ+MT group. Because glutathione (GSH) is the predominant antioxidant system in mammalian oocytes, we next detected intracellular GSH in different groups. We found that the GSH level was significantly decreased in oocytes exposed to OBZ (P < 0.05), while MT supplementation significantly increased the GSH level (Figure 4A, 4C). Furthermore, the expression levels of antioxidative stress genes (Sod1 and Gpx1) were detected by qRT-PCR. Remarkably, OBZ-exposed oocytes decreased the expression of antioxidative stress genes; however, the downregulation could be significantly reversed to a level comparable to that of the control group with MT treatment (Figure 3B).

Abnormal ROS and GSH levels could induce the occurrence of early apoptosis in oocytes, and we examined early apoptosis by Annexin-V staining assay in different groups. Our results showed that the percentage of Annexin-V-positive oocytes in the OBZ exposure group significantly increased compared with that in the control group (P < 0.05), while the increased percentage was significantly reversed by MT supplementation (Figure 4D, 4E). Moreover, we detected the relative mRNA expression of apoptosis-related genes in different groups. As shown by qRT-PCR analysis, OBZ exposure significantly downregulated the expression of the anti-apoptosis gene Bcl2 (Figure 3C), but it exhibited significant upregulation of pro-apoptosis genes, including Bax, Caspase-3, and P53 (Figure 3D). These transcriptional changes were reversed when MT was added to the IVM medium with OBZ.

### Effects of melatonin on mitochondrial characterization in mouse oocytes exposed to OBZ

**Figure 5:**
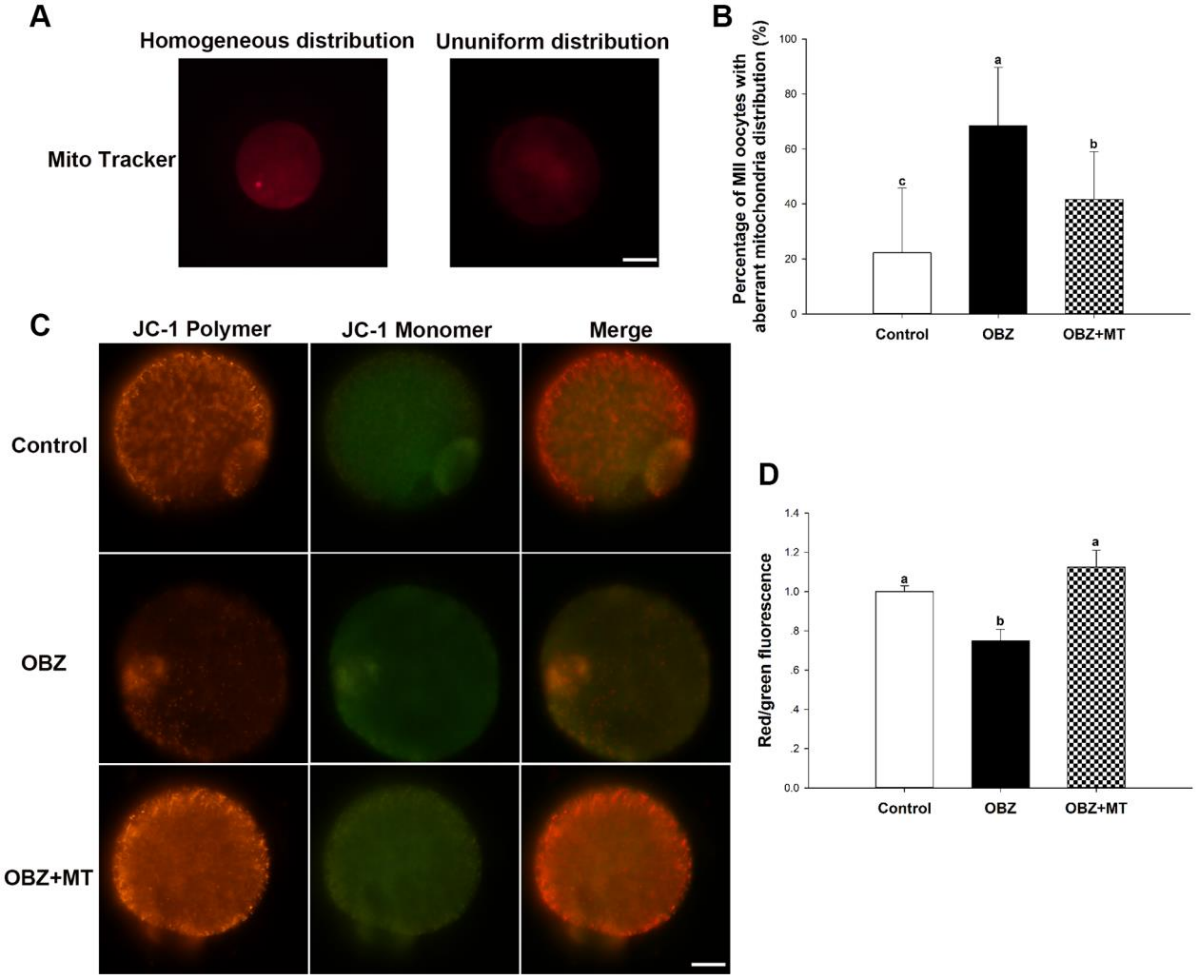
**Effects of melatonin on the mitochondrial distribution pattern and mitochondrial membrane potential (ΔΨm) in oxybenzone-exposed mouse oocytes.** (**A**) Representative images of homogeneous and nonuniform distribution patterns of mitochondria in oocytes. Scale bar, 30 μm. (**B**) Proportion of oocytes with aberrant distribution of mitochondria in each treatment group. (**C**) Representative images depicting JC-1 in the control, OBZ-exposed, and melatonin+OBZ-treated oocytes. Scale bar, 20 μm. (**D**) Relative ΔΨm represented as the ratio of red to green intensity. Values indicated by different letters are significantly different (P < 0.05). The experiments were repeated three times, with n = 15-20 per group. Control, untreated control group; OBZ, oxybenzone-exposed group; OBZ+MT, “oxybenzone + melatonin” treatment group.

Mitochondrial distributions are critical indicators of the cytoplasmic maturation of oocytes, so we then examined their changes in different groups. Oocytes exposed to OBZ showed a significantly increased percentage of maturated oocytes with aberrant mitochondrial distribution, while treatment with MT led to a significant improvement in the homogeneous distribution of mitochondria (Figure 5A, 5B).

Next, we investigated the mitochondrial ΔΨm using a mitochondrial membrane potential assay kit for detecting JC-1. The ratio of the intensity of rhodamine isothiocyanate to fluorescein isothiocyanate fluorescence, which reflects the mitochondrial ΔΨm, was significantly decreased in mouse oocytes exposed to OBZ compared to the control (P < 0.05), whereas the reduction could be significantly reversed to a level comparable to that of the control group with MT treatment (Figure 5C, 5D). In addition, the immunofluorescence staining results showed that OBZ exposure significantly decreased the average fluorescence intensity of ATP5A compared to the untreated group (Figure 2A, 2D). As expected, MT supplementation could restore the abnormal global levels of ATP5A. However, no statistically significant differences were found in the average fluorescence intensity of ATP5B among the control, OBZ, and OBZ+MT groups (Figure 2A, 2E).

### Effects of melatonin on spindle configuration in mouse oocytes exposed to OBZ

**Figure 6:**
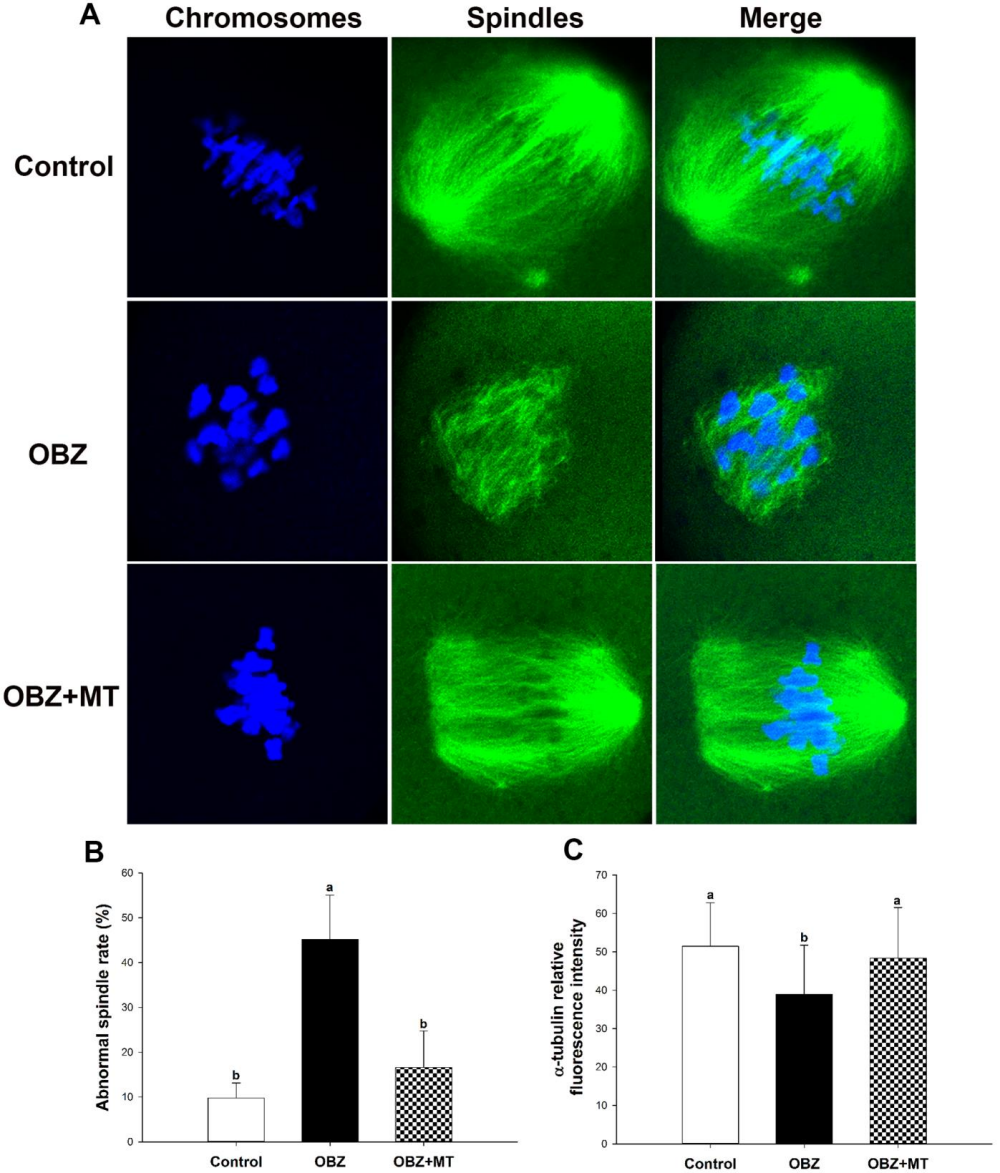
**Effects of melatonin on spindle formation and chromosome alignment in OBZ-exposed oocytes.** (**A**) Representative images of spindle morphologies and chromosome alignment in the control, OBZ-exposed, and melatonin+OBZ-treated oocytes. Green, α-tubulin; blue, DNA. (**B**) The rate of abnormal spindles in each treatment group. (**C**) Fluorescence intensity analysis of α-tubulin in the control, OBZ-exposed, and melatonin+OBZ-treated oocytes. Values indicated by different letters are significantly different (P < 0.05). The experiments were repeated five times, with n = 10-15 per group. Control, untreated control group; OBZ, oxybenzone-exposed group; OBZ+MT, “oxybenzone + melatonin” treatment group.

As meiotic arrest is largely due to abnormal spindle assembly, we then examined spindle morphologies by immunostaining with anti-α-tubulin-FITC antibody, and chromosome alignment was visualized by counterstaining with DAPI. Our results showed that most oocytes in the control group exhibited a typical barrel-shaped spindle apparatus with well-aligned chromosomes on the equatorial plate; however, spindle formation was severely disrupted, and chromosomes were disorganized in the OBZ exposure group, while melatonin supplementation relieved the disruption caused by OBZ (Figure 6A). The proportion of oocytes with aberrant spindle morphology in the OBZ-treated group was significantly higher than that in the control group (P < 0.05), while there was no difference between the OBZ+MT group and the control group (Figure 6B). Furthermore, we found that the average intensity of fluorescence labeling of α-tubulin in the OBZ-exposed oocytes was much weaker than it was in the untreated oocytes (P < 0.05), while there was no statistically significant difference between the control group and OBZ+MT group (Figure 6C).

### Effects of melatonin on *in vivo* development of OBZ-exposed mouse oocytes and embryos

**Figure 7:**
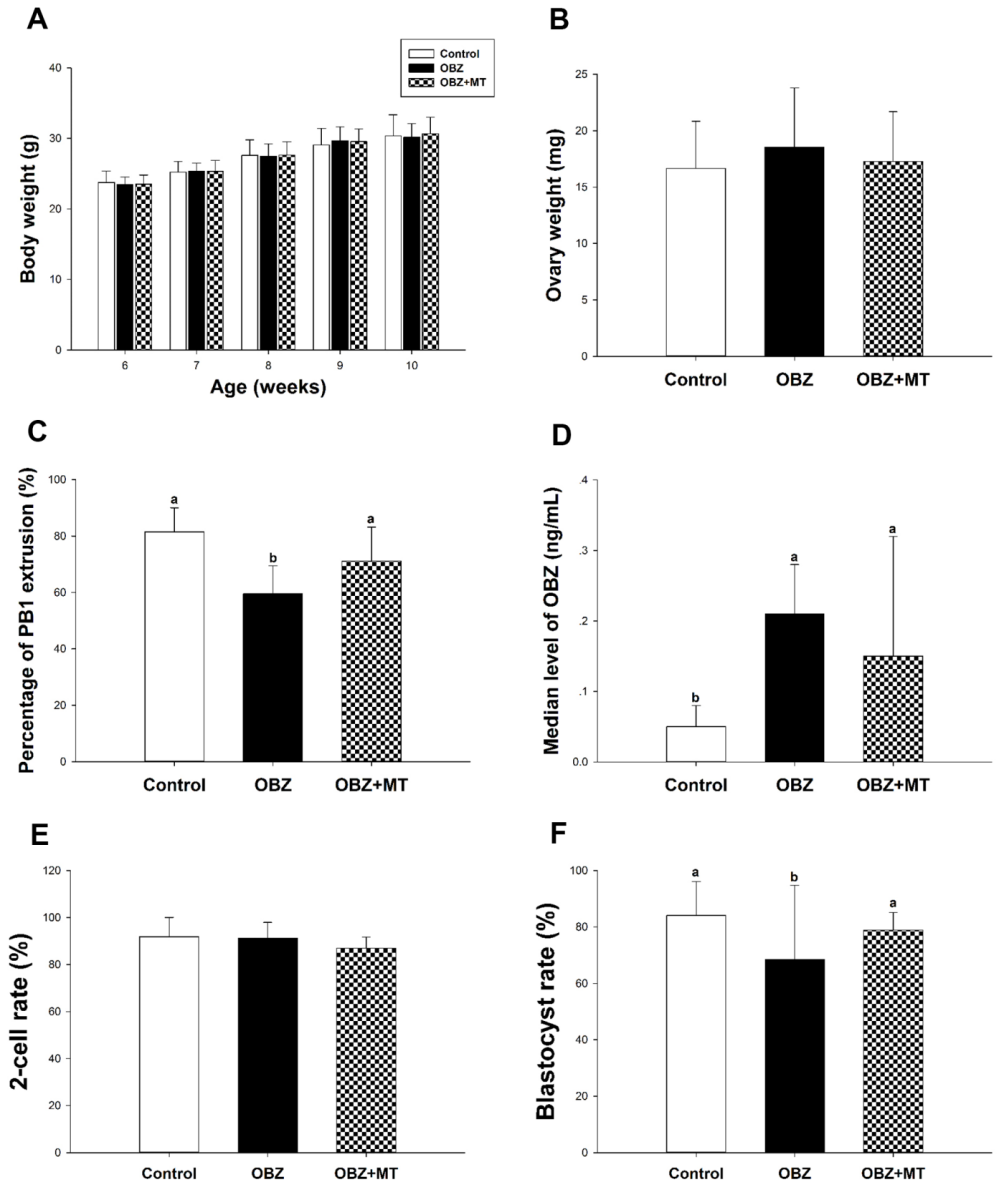
**Effects of melatonin on body weight, ovary weight, the rate of PBE, the concentration of OBZ, and embryo development from OBZ-exposed mice *in vivo***. All parameters were measured throughout the experimental period (6–10 weeks). ****(**A**–**F**) Body weight (**A**), ovary weight (**B**), extrusion rate of PB1 (**C**), concentrations of OBZ (**D**), and embryo development rate (**E**, **F**) from the 2-cell stage to blastocyst stage in each treatment group. Values indicated by different letters are significantly different (P < 0.05). Control, untreated control group; OBZ, oxybenzone-exposed group; OBZ+MT, “oxybenzone + melatonin” treatment group.

To corroborate the *in vitro* data, we then performed *in vivo* experiments. Our results showed that after 28 days of OBZ treatment with or without MT, no statistically significant differences were found in body weight or ovary weight among the different groups (Figure 7A, 7B). We then investigated for the first time the meiotic maturation of oocytes acquired from *in vivo* experiments. Similar to the IVM results, OBZ exposure in mice significantly decreased the PBE rate of oocytes compared with the control oocytes (59.58 ± 9.92%, n = 301 vs 81.51 ± 8.51%, n = 252, P < 0.05), while MT exposure significantly reversed oocyte maturation to a level that was comparable to that of the control group (71.16 ± 12.01%, n = 267, P = 0.26; Figure 7C). Next, we detected the whole blood concentrations of OBZ in different groups. As shown in Figure 7D, the average level of OBZ in the OBZ group was significantly higher than that in the control group (0.21 ± 0.07 ng/mL, n = 5 vs 0.05 ± 0.03 ng/mL, n = 5, P < 0.05), but it was similar to that of the OBZ+MT group (0.15 ± 0.07 ng/mL, n = 5). Intriguingly, the median concentration of OBZ in the control group was significantly lower than it was in the OBZ+MT group.

Next, we explored the early embryo developmental potential of embryos acquired from *in vivo* experiments. As shown in Figure 7F, the percentage of mouse embryos that reached the blastocyst stage was significantly lower in the OBZ group than it was in the OBZ+MT and control groups (68.53 ± 26.25%, n = 180 vs 84.10 ± 11.98%, n = 183; 78.89 ± 6.29%, n = 186, P < 0.05). However, the cleavage rates were similar in the three different groups (87.01 – 91.90%; Figure 7E).

### Effects of melatonin on oocyte quality in OBZ-exposed mouse models

**Figure 8:**
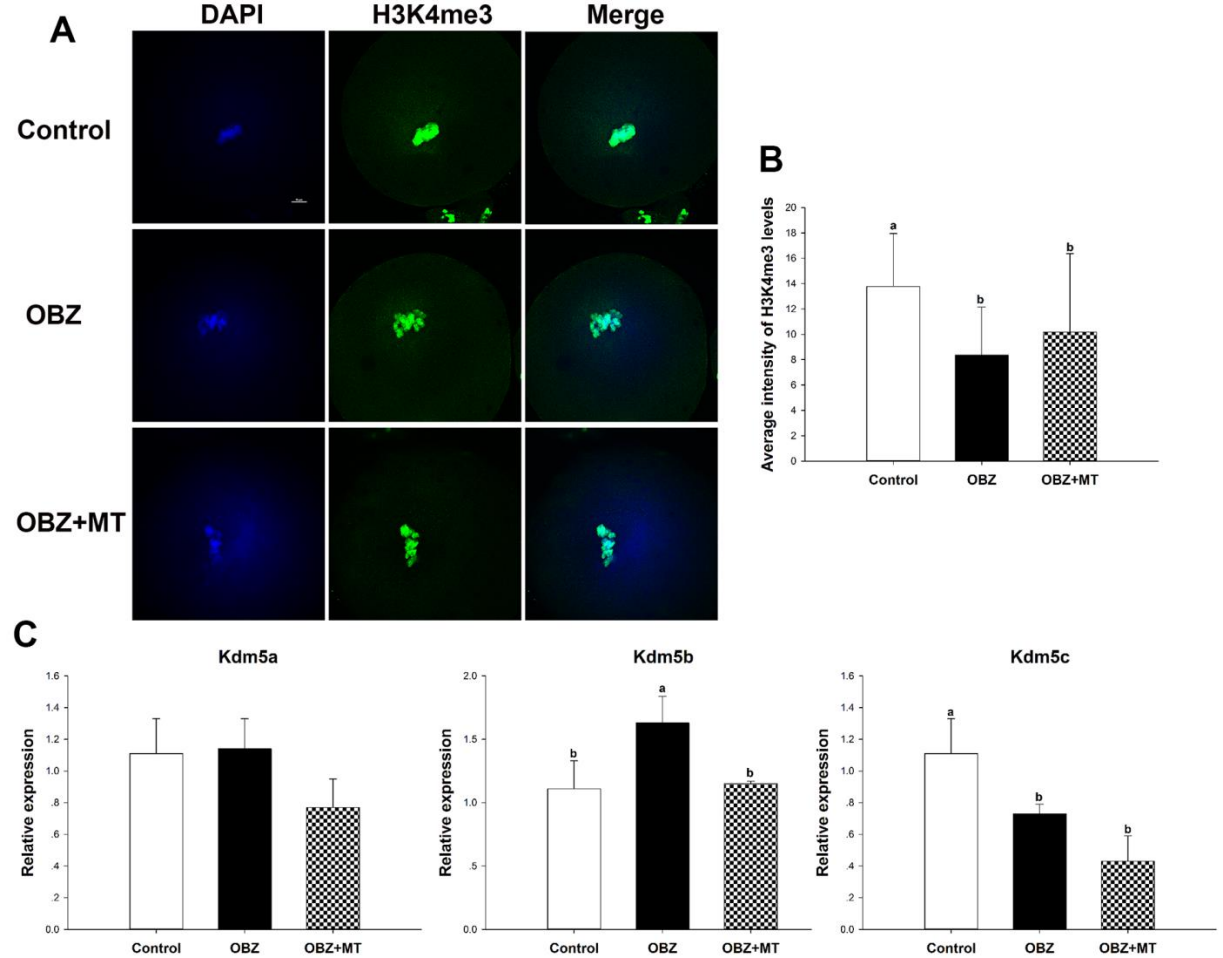
**Effects of melatonin on the H3K4me3 levels and expression of the Kdm5 family of genes in OBZ-exposed mice *in vivo*.** (**A**) Immunofluorescence staining for H3K4me3 in the control, OBZ-exposed, and melatonin+OBZ-treated oocytes. Green, H3K4me3; blue, DNA. Scale bar, 10 μm. (**B**) Average fluorescence intensity for H3K4me3 in mouse oocytes from the different groups. (**C**) Expression analysis of genes involved in histone H3K4me3 demethylation (Kdm5a, Kdm5b, and Kdm5c) from different groups. Values indicated by different letters are significantly different (P < 0.05). The experiments were repeated five times, with n = 10-15 per group. Control, untreated control group; OBZ, oxybenzone-exposed group; OBZ+MT, “oxybenzone + melatonin” treatment group.

We then asked whether OBZ administration could affect histone H3K4me3 in *in vivo* matured oocytes. As shown in Figure 8A, the level of histone H3K4me3 in the OBZ group was also significantly decreased compared with that of the control group (P < 0.05). In contrast with the *in vitro* results, MT administration did not significantly improve the global histone H3K4me3 level compared with that of the OBZ group (Figure 8A, 8B). Moreover, histone H3K4me3 demethylases were investigated by qRT-PCR. Our results showed that there was no difference in Kdm5a mRNA expression among the three different groups (Figure 8C). In the OBZ group, the Kdm5b transcript level was significantly increased compared with that in the control group (P < 0.05), while the abnormal increase could be reversed by melatonin administration (Figure 8C). Unexpectedly, the expression of Kdm5c was significantly downregulated in the OBZ and OBZ+MT groups (Figure 8C).

**Figure 9:**
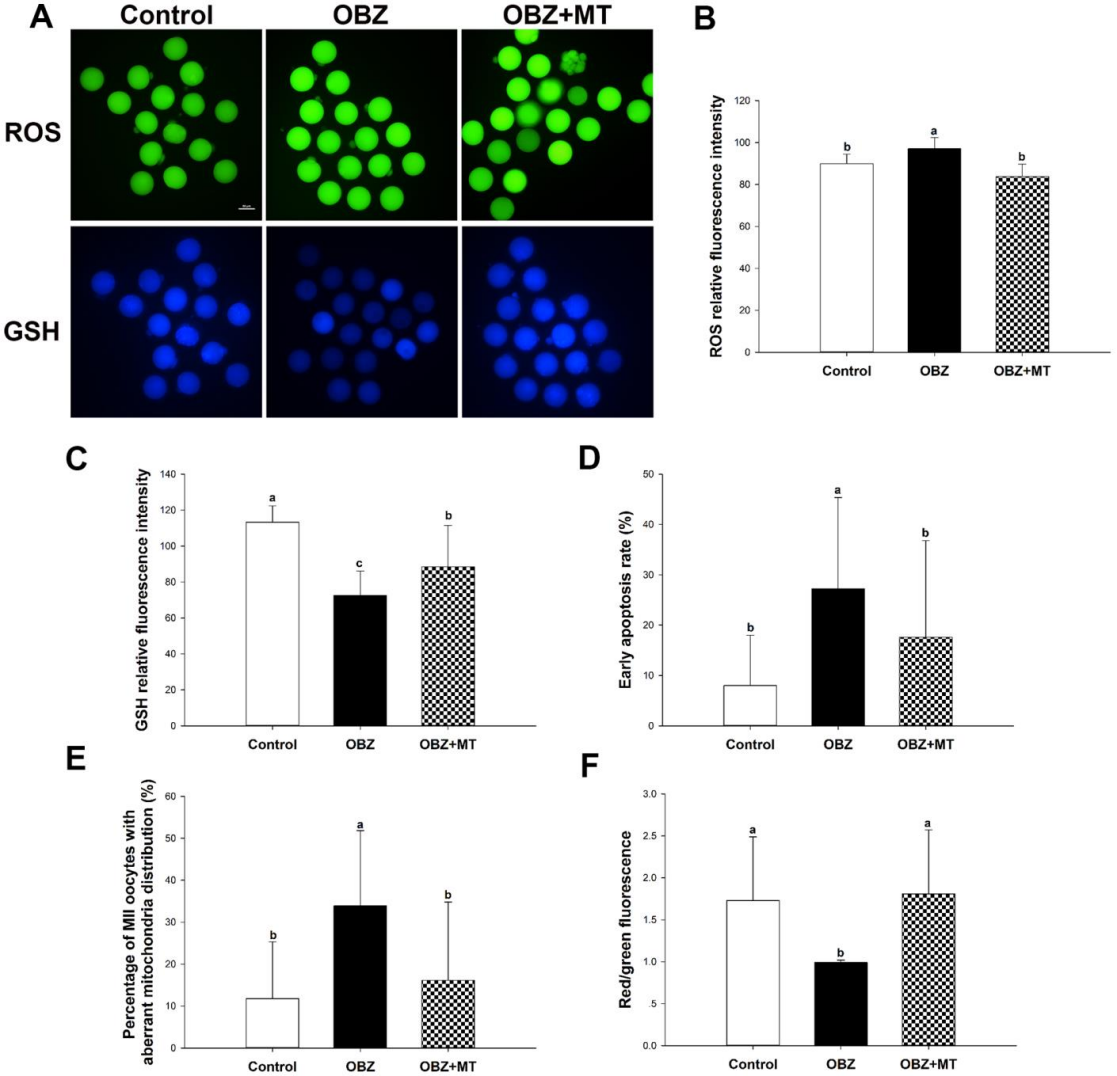
**Effects of melatonin on the quality of matured oocytes from OBZ-exposed mice *in vivo*.** (**A**) Representative images of ROS and GSH levels in the control, OBZ-exposed, and melatonin+OBZ-treated oocytes. Scale bar, 50 μm. (**B**) Intracellular ROS and (**C**) GSH levels in mouse oocytes from each treatment group. (**D**) Proportion of early apoptotic oocytes in each treatment group. (**E**) Proportion of oocytes with aberrant distribution of mitochondria in each treatment group. (**F**) Relative MMP is represented as the ratio of red to green intensity. Values indicated by different letters are significantly different (P < 0.05). The experiments were repeated three times, with n = 15-20 per group. Control, untreated control group; OBZ, oxybenzone-exposed group; OBZ+MT, “oxybenzone + melatonin” treatment group.

To further verify the results of *in vitro* experiments, the intracellular ROS levels, GSH levels, early apoptotic phenomena, and mitochondrial characterization were investigated. In the OBZ group, the ROS signal (Figure 9A, 9B) and rate of early apoptosis in oocytes (Figure 9A, 9D) were markedly increased compared to the control (P < 0.05), while the level of GSH was significantly decreased (P < 0.05; Figure 9A, 9C). As expected, MT significantly counteracted oxidative damage and inhibited early apoptosis (Figure 9A–9D). The rate of oocytes with aberrant mitochondrial distribution was not only increased in the OBZ group (P < 0.05; Figure 9E), but the mitochondrial ΔΨm was significantly decreased when compared with their controls (P < 0.05; Figure 9F). Similar to the *in vitro* results, melatonin administration significantly rescued aberrant mitochondrial characterization (Figure 9E, 9F).

**Figure 10:**
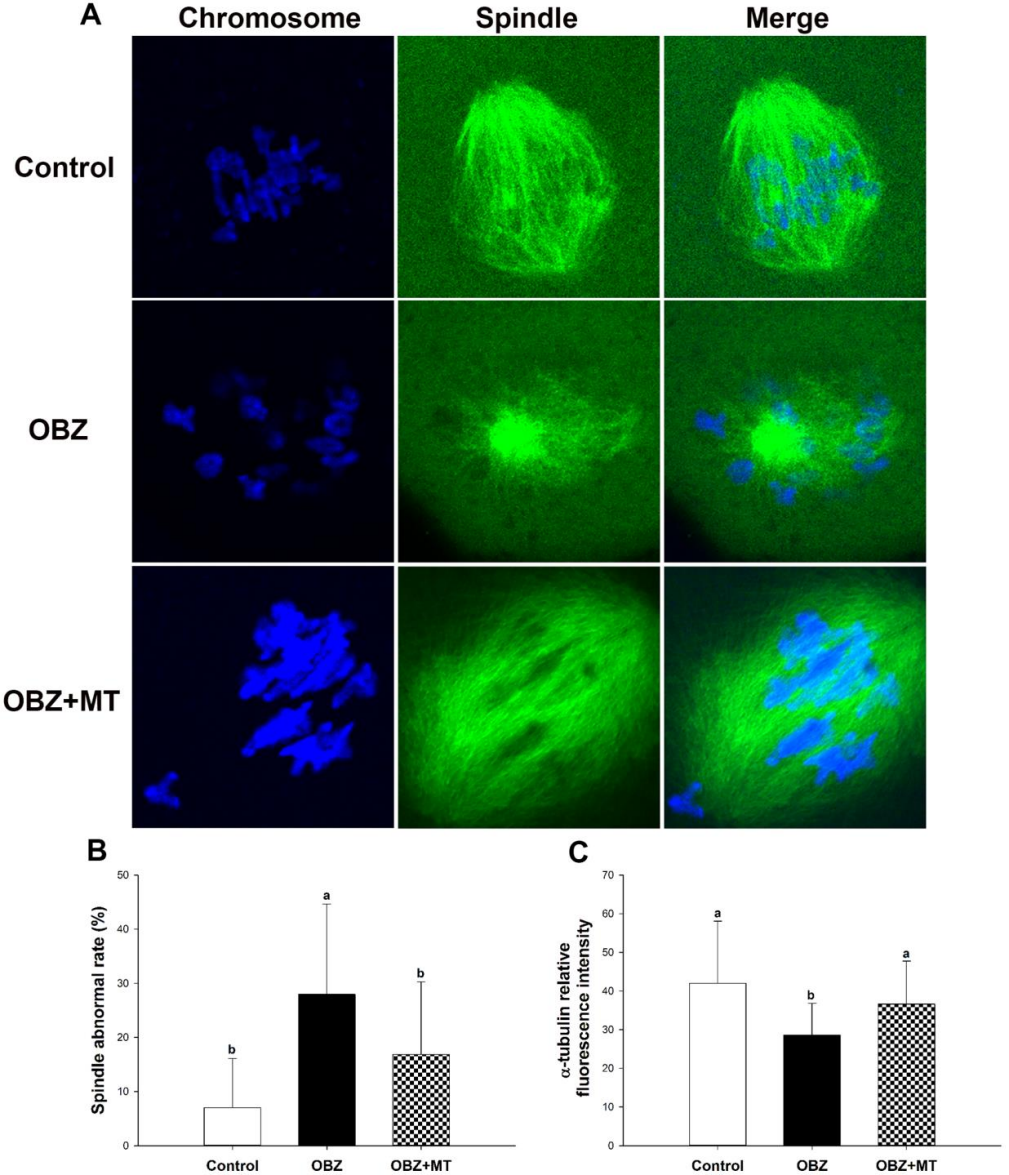
**Effects of melatonin on spindle formation and chromosome alignment in OBZ-exposed mice *in vivo*.** (**A**) Representative images of spindle morphologies and chromosome alignment in the control, OBZ-exposed, and melatonin+OBZ-treated oocytes. Green, α-tubulin; blue, DNA. (**B**) The rate of abnormal spindles observed in each treatment group. (**C**) Fluorescence intensity analysis of α-tubulin in the control, OBZ-exposed, and melatonin+OBZ-treated oocytes. Values indicated by different letters are significantly different (P < 0.05). The experiments were repeated three times, with n = 15-20 per group. Control, untreated control group; OBZ, oxybenzone-exposed group; OBZ+MT, “oxybenzone + melatonin” treatment group.

Next, we investigated spindle morphologies and chromosome alignment through immunofluorescence staining. Our results showed that control oocytes usually exhibit a typical barrel-shaped spindle and well-aligned chromosomes at the equator (Figure 10A). In contrast, many disorganized spindle morphologies and misaligned chromosomes were observed in the OBZ group (Figure 10A). We found a higher percentage of spindle defects and chromosome congression failure in OBZ-treated oocytes than we found in their controls (P < 0.05; Figure 10B). Moreover, the average fluorescence intensity of α-tubulin in the untreated group was much higher than it was in the OBZ group (P < 0.05; Figure 10C). It was noted that the defects of the spindle could be restored by MT treatment (Figure 10A–10C).

## DISCUSSION

OBZ, an organic UV filter, is widely used in sunscreens, personal care products, and food product packaging, and it is found almost everywhere in the environment [11]. Furthermore, OBZ is small enough to allow it to penetrate through skin and be detected in urine, breast milk, and blood [8]. Several lines of investigation have revealed that OBZ is an EDC that adversely impacts the embryonic development and fertilization of mammals [32], decreases gestational age in humans [11], causes apoptosis of neuronal cells [33], and is associated with neonates born with HSCR [21]. Therefore, an increasing number of people are paying attention to the toxicological effects of OBZ on the reproductive system and looking for ways to alleviate OBZ-induced impairments.

In the present study, we aimed to explore the potential detrimental effects of OBZ on mouse oocyte maturation and determine the protective effects of MT on OBZ-exposed oocytes. Our study provided the first evidence that OBZ reduced the rate of PBE in a dose-dependent manner, suggesting that OBZ exposure perturbs normal meiotic progression. In addition, the blastocyst formation rate was significantly decreased by OBZ exposure. Similar effects were also reported from previous studies in OBZ-exposed mouse neuronal cells and human 293T cells [21, 33]. It is noteworthy that the human plasma concentration of OBZ ranged from 740 nM to 920 nM, while the FDA safety threshold is 0.5 ng/mL (approximately 2.2 nM) [10]. However, our data from an *in vitro* study showed that more than 25 nM OBZ exposure led to a significant decrease in the percentage of oocytes extruding the polar body (Figure 1), revealing the need for additional studies investigating the safety of OBZ. In our *in vivo* study, OBZ exposure did not affect body weight or ovary weight in mice. Interestingly, the mean whole blood concentration of OBZ in OBZ-exposed mice was 0.21 ng/mL, which fell below the threshold, but the PBE rate of oocytes in the OBZ exposure group was significantly lower than that in the control group. This observation implies that mice may be more susceptible to OBZ exposure than humans, but that requires further investigation. Research within the last decade has found that MT plays a key role in a variety of physiological functions in tissues and cell types, including ovarian function, oocyte maturation, and embryonic development [34, 35]. In ART, MT would be expected to elevate the pregnancy rate by improving the quality of oocytes as well as promoting the fertilization rate and embryo development [30]. We thus propose that MT can ameliorate OBZ-induced oocyte meiotic failure and decreases in embryonic developmental competence. As expected, the detrimental effects of OBZ on oocyte maturation and early embryo development could be rescued by treatment with low concentrations of exogenous MT (10^-7^ M). Moreover, in line with previous studies, the appropriate concentration of MT (10^-7^ M) supplementation could alleviate ochratoxin A and paraquat-induced toxicity in porcine and bovine oocytes [24, 35]. These results were also confirmed by our *in vivo* experiments.

Histone modifications are highly dynamic and regulate the expression of developmental genes during oogenesis and embryogenesis in mammals [36]. Research within the last decade has found distinct H3K4me3 landscapes during mouse oocyte growth and zygotic genome activation (ZGA) [37–39]. Some broad, noncanonical forms of H3K4me3 (exceeding 5 kb) were widely observed in full-grown and mature oocytes. ZGA during the two-cell stage is associated with re-establishment of canonical H3K4me3 marks, which are restricted to the transcription start site of transcriptionally active genes. Unlike in mouse oocytes, H3K4me3 largely exhibits canonical patterns at promoters in human oocytes [40]. After fertilization, pre-ZGA embryos acquire permissive chromatin and widespread H3K4me3 in CpG-rich regulatory regions. Therefore, the distribution of H3K4me3 plays critical roles in oocyte meiotic maturation and subsequent embryo development. In the present study, we found that OBZ exposure significantly decreased the average fluorescence intensity of H3K4me3 and increased the transcript levels of Kdm5b, which is capable of demethylating tri-, di- and monomethylated lysine 4 of histone H3. These findings are in line with previous studies showing that aberrant Kdm5b and H3K4me3 levels could affect oocyte maturation and meiosis resumption [41, 42]. A previous study [43] showed that poly (ADP-ribose) polymerase-1 (PARP-1) knockdown increased Kdm5b levels and decreased H3K4me3 levels at the promoters of the positively regulated genes in MCF-7 cells. PARP-1 shows a dynamic association with distinct nuclear compartments in the GV of pre-ovulatory oocytes and upon meiotic resumption, with critical components of the meiotic spindle as well as pericentric heterochromatin domains in mature metaphase II eggs [44]. Thus, MT prevented OBZ-induced increase in Kdm5b expression and demethylation of H3K4me3 might associate with reduction of PARP-1 expression. This was consistent with our *in vivo* experiments and previous studies, which showed that MT rescued abnormal epigenetic alterations in EDC-exposed oocytes [35, 45].

Accumulated physiological levels of ROS lead to oxidative stress, which could affect the biology of female reproduction by targeting ovaries, follicles, and oocytes [46]. Activation of intracellular ROS is highly associated with OBZ-induced toxicity in many cell types [33, 47]. Our results showed that OBZ exposure significantly increased ROS levels and decreased GSH levels compared with those of the control. Furthermore, the expression of Sod1 and Gpx1 decreased in the OBZ-treated group. As Sod1 and Gpx1 play important roles in protecting cells from oxidative damage [48], our results demonstrated that OBZ promoted oxidative stress during mouse oocyte maturation. A recent study demonstrated that a deficiency in fibroblast peroxisome proliferator-activated receptors (PPARs) increased oxidative stress in wound microenvironment [49]. It is reported that ROS triggers activation of ERK1/2, PDGF, and PI2K/Akt resulting in increased transcription of PPARs [50]. Moreover, PPARs play a protective role in cardiovascular function by increasing activities of antioxidant genes, such as Sod1, Sod2, and catalase [51]. It is worth noting that melatonin treatment significantly increases the expression of PPAR in mice [52], rats [53], and different cells [52, 54], which indicates the intertwine between transcription regulator and circadian clocks. Therefore, the protective roles of melatonin against toxicity of oxybenzone exposure during *in vitro* and *in vivo* maturation of mouse oocytes might be associated with PPAR signaling pathway to reduce ROS production.

A recent publication by Bazopoulou et al. (2019) showed that global H3K4me3 levels are ROS-sensitive and that depletion of H3K4me3 levels increases stress resistance in mammalian cell cultures [55]. Thus, demethylation of H3K4me3 in OBZ-exposed oocytes may combat oxidative stress. In addition, oxidative stress leads to apoptosis, which is generally activated by EDCs, such as ochratoxin A [24], bisphenol A [29], and aflatoxin B1 [56]. We then examined early apoptosis in oocytes through the use of Annexin-V staining, which has a high affinity for phosphatidylserine. The results showed that the early apoptosis levels in the OBZ group were higher than those in the control group. Furthermore, OBZ significantly upregulated Bax, Caspase-3, and P53 and downregulated Bcl2 gene expression (Figure 3). Likewise, MT treatment increased the expression of Bcl2 and decreased the transcript levels of Bax, Caspase-3, and P53, suggesting that MT blocked early apoptosis in OBZ-treated oocytes. This is consistent with the findings reported in previous studies [45, 57]. Similar to our *in vitro* study, MT ameliorated oxidative stress and early apoptosis *in vivo*.

Mitochondria, which are the primary sites of melatonin synthesis, play critical roles in fundamental cellular processes, energy production, and oxidative balance. Melatonin receptor 1 (MT1) were identified in the mitochondrial membrane and loss of the MT1 enhanced neuronal vulnerability and potentially accelerated the neurodegenerative process [58]. In another study conducted by Onphachanh et al. (2017) [59], melatonin receptor 2 (MT2) improved neuronal cell survival in response to high-glucose insult through PINK1 activation, stimulation of Akt phosphorylation, and resultant preservation of mitochondrial function. Melatonin treatment or MT2 overexpression suppressed the activation of markers of endoplasmic reticulum stress (cleaved caspase-12 and CHOP) and mitochondria (cleaved caspase-9 and cytochrome c) without affecting cleaved caspase-8 activity [60]. It is noteworthy that MT2 mutation is associated with increased type 2 diabetes risk and obese [61, 62]. Considering the fact that diabetes and obesity is a well-known risk factor for Polycystic ovary syndrome, MT2 might be regarded as a potential therapeutic target when designing clinical trials to examine melatonin-based therapy for oocyte maturation failure. In this study, MitoTracker and mitochondrial membrane potential assays were utilized to explore the distribution of mitochondria and the mitochondrial membrane potential state in oocytes. Our data from *in vitro* and *in vivo* studies revealed that OBZ exposure impaired the uniform distribution of mitochondria and decreased mitochondrial ΔΨm, whereas MT could rescue OBZ-induced mitochondrial defects. These findings are in line with previous studies showing that MT supplementation improved mitochondrial quality during oocyte maturation [24, 63]. However, whether MT1 and MT2 are involved in the protective effects of melatonin on OBZ-exposed oocytes requires further investigation. Mitochondria have been shown to affect spindle alignment in higher eukaryotes [64], and proper spindle formation is important for the orchestration of meiotic progression. In the present study, we found that OBZ exposure adversely influenced spindle configuration and chromosome alignment. In studies using oocytes from mice and pigs, it has been reported that impaired spindle assembly and alignment can be restored upon *in vitro* incubation of oocytes in culture medium supplemented with MT [29, 56]. As expected, treatment with MT improved the quality of oocytes by reducing the frequency of spindle defects. These observations were also confirmed by our *in vivo* experiments.

To the best of our knowledge, this is the first report to confirm that MT has a protective effect against OBZ-induced deterioration of oocytes during their maturation. We found that OBZ exposure altered histone H3K4 methylation, induced oxidative stress and apoptosis, caused mitochondrial dysfunction, and impaired spindle assembly, which further influenced the maturation of mouse oocytes *in vitro* and *in vivo*. By applying an appropriate level of MT, we found that the OBZ-induced adverse effects can be effectively reversed. Our study provides insights into the potential mechanisms underlying the toxic effects of OBZ and identified a potential means for melatonin to reduce OBZ-induced damage in oocytes, which may provide a promising reference for further studies to consider the safety threshold of OBZ.

## MATERIALS AND METHODS

### Mice and chemicals

Institute of Cancer Research (ICR) mice were used in this study. All experiments were approved by the Animal Ethics Committee of the Third Affiliated Hospital of Guangzhou Medical University and were performed in accordance with the guidelines of Animal Care and Use of the Third Affiliated Hospital of Guangzhou Medical University (TAHGMUACC-2019-0126).

Unless otherwise specified, all chemicals and reagents used in this study were purchased from Sigma Chemical Company (St. Louis, MO, USA). OBZ was purchased from Selleck Chemicals (Houston, TX, USA). A rabbit anti-H3K4me3 antibody and a rabbit anti-H3K36me3 antibody were purchased from Cell Signaling Technology (Devers, MA, USA). Mouse anti-ATP5A and mouse anti-ATP5B antibodies were purchased from Santa Cruz (Santa Cruz, CA, USA). Alexa Fluor 488 goat anti-rabbit and Alexa Fluor 594 goat anti-mouse antibodies were purchased from Invitrogen (Carlsbad, CA, USA).

### Oocyte/zygote collection and culture

For the *in vitro* experiment, 6- to 10-week-old female ICR mice were intraperitoneally injected with 5 international units (IU) of pregnant mare serum gonadotropin (PMSG). Forty-six to 48 hours later, oocytes with intact germinal vesicle were collected from the ovaries of mice, washed three times in M2 medium, and then cultured in M16 medium under paraffin oil at 37° C in a humidified incubator with 5% CO_2_, 5% O_2_, and 90% N_2_.

For the *in vivo* experiment, 90 female mice (6 weeks old) from the ICR were distributed equally into 3 groups at random: control, OBZ (drinking water with 500 nM OBZ per day for 28 days), OBZ+MT (drinking water with 500 nM OBZ and MT 15 mg/kg body weight per day for 28 days). For metaphase II (MII) oocyte collection, mice were superovulated by the injection of 5 IU of PMSG, which was followed by administration of 5 IU of human chorionic gonadotropin (hCG) after 48 hours. Cumulus-oocyte complexes were collected from the oviduct ampullae 14 to 16 hours post-hCG, and denuded MII oocytes were obtained by removing the cumulus mass in medium containing 0.5 mg/mL hyaluronidase, which took place for 2 min at 37° C in a 5% CO_2_ atmosphere.

For zygote collection, ICR females were primed with intraperitoneal injection of 5 IU each if PMSG and hCG 48 h apart. The superovulated female mice were mixed with males in the same cage at a 1:1 ratio. Zygotes were collected from oviducts and were cultured in KSOM medium at 37° C in a humidified incubator with 5% CO_2_, 5% O_2_, and 90% N_2_.

### OBZ and MT treatment

OBZ was dissolved in DMSO to produce a 50 mmol/L stock solution and then was diluted in M16 medium to produce final concentrations of 2, 50, 250, 500, and 1000 nmol/L. The final concentration of DMSO was less than 0.1% of the culture medium.

Melatonin was dissolved in absolute ethanol to 0.1 mol/L and then diluted in 500 nmol/L OBZ-containing M16 medium to yield final concentrations of 1 × 10^−9^ mol/L, 1 × 10^−7^ mol/L, and 1 × 10^−5^ mol/L. The final concentration of ethanol was less than 0.1% of the culture medium.

### Immunofluorescence staining

Mouse oocytes were washed three times in 1% PVA-supplemented PBS (PBS-PVA) and then were fixed with 4% paraformaldehyde in PBS (PBS-PFA) for 30 min. These oocytes were permeabilized using PBS containing 1% Triton X-100 for 20 min and then were blocked in PBS containing 2% bovine serum albumin (PBS-BSA) for 2 h at room temperature. After blocking in PBS-BSA, oocytes were stained by incubation with different primary antibodies (1:400 for H3K4me3; 1:800 for H3K36me3; 1:200 for ATP5A and ATP5B; and 1:300 for α-tubulin) at 4° C overnight. After extensive washing, the samples were then incubated with Alexa Fluor 488 goat anti-rabbit antibody (1:500) and Alexa Fluor 594 goat anti-mouse antibody at room temperature for 1 hour. Oocytes were counterstained with DAPI for 10 min after three washes in PBS-BSA. Finally, oocytes were mounted on glass slides and were viewed under a confocal laser scanning microscope (Nikon A1 R, Tokyo, Japan). The fluorescence intensities of oocytes were analyzed using ImageJ software (National Institutes of Health, Bethesda, MD, USA). Each experiment was repeated at least 3 times with at least 30 oocytes.

### Detection of reactive oxygen species (ROS), glutathione (GSH), and early apoptosis

Matured oocytes from different groups were analyzed for their intracellular ROS and GSH levels using 2’,7’-dichlorodihydrofluorescein diacetate (DCHFDA) and a ThiolTracker™ Violet GSH detection reagent (Invitrogen/Molecular Probes, Carlsbad, CA, USA), respectively. In brief, oocytes were incubated with 100 μmol/L DCHFDA or 20 μmol/L prewarmed ThiolTracker™ Violet dye solution in the dark for 30 min at 37° C, washed three times in PBS-PVA, and viewed under an epifluorescence microscope (Nikon, Tokyo, Japan). The fluorescence intensities of oocytes were analyzed using ImageJ software (National Institutes of Health, Bethesda, MD, USA). Each experiment was repeated at least 3 times with at least 30 oocytes.

The early apoptosis of mouse oocytes was evaluated by an Annexin-V-FITC kit (Life Technologies Inc., Grand Island, NY, USA). According to the manufacturer’s instructions, samples were washed with 1× annexin-binding buffer for 5 min and incubated in 490 μL 1× annexin-binding buffer supplemented with 10 μL annexin-V conjugate for 15 min at room temperature in the dark. Then, oocytes were moved to glass slides three times with PBS-PVA. The fluorescence signal of oocytes was examined immediately under an epifluorescence microscope (Nikon, Tokyo, Japan). Each experiment was repeated at least 3 times with at least 30 oocytes.

### Mitochondrial staining

Mitochondrial distribution was detected using MitoTracker Deep Red (Invitrogen, Paisley, UK). Matured oocytes were incubated with 200 nmol/L of MitoTracker probe in M2 medium for 30 min at 37° C. Then, oocytes were washed three times with PBS-PVA and fixed in PBS-PFA for 40 min at room temperature. Fluorescence signals were detected using an epifluorescence microscope (Nikon, Tokyo, Japan). Each experiment was repeated at least 3 times with at least 30 oocytes.

Mitochondrial membrane potential (ΔΨm) was determined using a MitoProbe™ JC-1 Assay Kit (Life Technologies Inc, Grand Island, NY, USA) according to the manufacturer’s instructions. Briefly, oocytes were incubated in M2 medium containing 4 μmol/L JC-1 (5,5’,6,6’-tetrachloro-1,1’,3,3’-tetraethylbenzimidazolyl-carbocyanine iodide) at 37° C for 30 min in the dark. The oocytes were then washed three times with PBS-PVA, mounted on slides under cover slips, and examined immediately under an epifluorescence microscope (Nikon, Tokyo, Japan). Microphotographs were analyzed using ImageJ software (National Institutes of Health, Bethesda, MD, USA). Mitochondria ΔΨm was expressed as the ratio of red fluorescence, which corresponded to activated mitochondria (J-aggregates), to green fluorescence, which corresponded to less-activated mitochondria (J-monomers). Each experiment was repeated at least 3 times with at least 30 oocytes.

### Measurement of OBZ concentration

Whole blood samples were collected into sterile BD Vacutainer^®^ plastic lithium heparin tubes and then were stored at 4° C until analysis. Total (free plus conjugated species) concentrations of OBZ in samples were measured using a previously described method [65]. In brief, whole blood samples were incubated in 1 mol/L ammonium acetate buffer solution for hydrolyzation with β-glucuronidase/sulfatase. After hydrolysis, phenols were extracted and preconcentrated with solid phase extraction and then were further detected using ultrahigh-performance liquid chromatography-electrospray ionization tandem mass spectrometry (Waters, Milford, MA, USA). The detection was performed in negative ion mode by multiple reaction monitoring. The limit of detection (LOD) was 0.04 ng/mL. Strict quality control was conducted during the analysis. CR data were collected using an automated chemistry analyzer (Hitachi 7020, Tokyo, Japan), which were obtained for correcting the phenol concentrations caused by blood concentration and dilution. The experiment for the detection of OBZ levels in each group was repeated 5 times.

### Quantitative real-time PCR (qRT-PCR)

To investigate the mRNA expression levels of histone H3K4me3 demethylation-related genes (Kdm5a, Kdm5b, and Kdm5c), antioxidative stress genes (SOD and GPx1), and apoptosis-related genes (Bcl2, Bax, Caspase-3, and P53), oocytes from different groups were collected. Glyceraldehyde 3-phosphate dehydrogenase (GAPDH) was used as an endogenous reference gene. Total RNA was extracted from the oocytes using a Qiagen RNeasy Mini Kit (Qiagen, Toronto, Canada); then, the RNA was reverse transcribed to generate cDNA and was stored at -20° C until use. Real-time PCR amplification was conducted with a One Step SYBR PrimeScript RT-PCR Kit (TaKaRa Bio. Inc., Tokyo, Japan) on an ABI-7900 SDS instrument (Applied Biosystems, Foster City, CA, USA). Each real-time PCR consisted of 10 μL of Advanced SYBR Green PCR Master Mix, 4.8 μL of nuclease-free water, 4 μL of cDNA sample, and 1.2 μL of gene-specific primers. The amplification protocol comprised an initial denaturation step at 95° C for 30 s, which was followed by 40 cycles of denaturation at 95° C for 5 s, annealing at 60° C for 30 s and extension at 72° C for 1 min. All oligonucleotide primer sequences are presented in Supplementary Table 1. The expression of each target gene was quantified relative to that of an internal reaction control gene (GAPDH) using the equation R = 2^-[ΔCt sample-ΔCt control]^. For ease of comparison, the average mRNA transcript level of each gene from the control group was set as one.

### Statistical analysis

For each treatment, there were at least 3 biological replicates, and no fewer than 30 oocytes were examined; results are expressed as the mean values ± standard error of the mean (SEM). The data were analyzed with univariate analysis of variance (ANOVA) followed by Tukey’s test using SPSS 17.0 statistical software (SPSS, Inc., Chicago, IL, USA). Differences in gene expression and fluorescence intensity were compared by Student’s t test. P values < 0.05 were regarded as statistically significant.

## Supplementary Material

Supplementary Table 1
