# Supplementary material for: Melatonin protects against oxybenzone-induced deterioration of mouse oocytes during maturation
Source: Aging (Albany NY). 2020 Dec 29;13(2):2727–49. doi: 10.18632/aging.202323 (PMC7880374; doi:10.18632/aging.202323)
Supplement: Supplementary Table 1 [file aging-13-202323-s001.pdf]

SUPPLEMENTARY TABLE

Supplementary Table 1. List of primers for qRT-PCR used in this study.

Gene name	Primer sequences (5'-3')	Product size (bp)	Accession number
p53	F: TGAGGTTCGTGTTTGTGCCTGC	165	XM_030245923.1
	R: CCATCAAGTGGTTTTTTCTTTTGC		
Bax	F: TTTGCTTCAGGGTTTCATCC	162	XM_011250780.3
	R: ATCCTCTGCAGCTCCATGTT		
Caspase-3	F: AAAGGCTGGAACCCTTGTTT	220	XM_030243266.1
	R: GCACCTTGCCTTCAATGAGT		
Bcl2	F: TCGCTACCGTCGTGACTTCGC	273	NM_009741.5
	R: GCATCCCAGCCTCCGTTATCC		
Sod1	F: CACTCTCAGGAGAGCATTCCA	110	XM_021185540.2
	R: CCCAGCATTTCCAGTCTTTG		
Gpx1	F: CCAGGAGAATGGCAAGAATGAA	138	XM_021207183.2
	R: AGGAAGGTAAAGAGCGGGTGAG		
Kdm5a	F: CCAGCCTTTCTACCCAATGT	146	XM_029478250.1
	R: ATAGGGCGTCCTTTAGCAGA		
Kdm5b	F: AAGCCAAGCTCTGTTCAGCAA	73	XM_017312617.2
	R: GAAGGCAATCGTTCTTCTCACT		
Kdm5c	F: GGCTGACATCCATTCCAAAG	88	XM_017318443.2
	R: AGCATACTCCTCCTCCTCTG		
GAPDH	F: CAGGTTGTCTCCTGCGACTTCA	190	NM_001289726.1
	R: GTGGGTGGTCCAGGGTTTCTTA		
